# Overexpression of ABCG2 Confers Resistance to MLN7243, a Ubiquitin-Activating Enzyme (UAE) Inhibitor

**DOI:** 10.3389/fcell.2021.697927

**Published:** 2021-07-14

**Authors:** Zhuo-Xun Wu, Yuqi Yang, Jing-Quan Wang, Silpa Narayanan, Zi-Ning Lei, Qiu-Xu Teng, Leli Zeng, Zhe-Sheng Chen

**Affiliations:** ^1^Department of Pharmaceutical Sciences, College of Pharmacy and Health Sciences, St. John’s University, Queens, NY, United States; ^2^Precision Medicine Center, The Seventh Affiliated Hospital, Sun Yat-sen University, Shenzhen, China

**Keywords:** multidrug resistance, MLN7243, transported substrate, ATP-binding cassette transporters, ABCG2

## Abstract

Overexpression of ATP-binding cassette transporter superfamily G member 2 (ABCG2), is known as a major mechanism mediating multidrug resistance (MDR) in cancer cells. MLN7243 is a small-molecule ubiquitin activating enzyme inhibitor currently under clinical investigation. The aim of the current study is to determine if MLN7243 is a substrate of MDR-related ABCG2 transporter. Our results showed that cancer cells overexpressing ABCG2 transporter were resistant to MLN7243 compared to the parental cells, while knockout of ABCG2 gene or pharmacological inhibition of ABCG2 efflux function completely reversed the drug resistance. Unexpectedly, the endogenous low expression of ABCG2 is sufficient to confer cancer cells resistance to MLN7243. The ABCG2 ATPase assay and HPLC assay suggested that MLN7243 can significantly stimulate ABCG2 ATPase activity and be pumped out from ABCG2-overexpressing cells by ABCG2. The docking analysis also implied that MLN7243 binds to ABCG2 drug-binding pocket with optimal binding affinity. However, MLN7243 did not competitively inhibit the efflux of other ABCG2 substrate drugs, indicating it may not serve as an MDR reversal agent. In conclusion, our study provides direct *in vitro* evidence to show that MLN7243 is a potent ABCG2 substrate. If our results can be translated to humans, it suggests that combining MLN7243 with ABCG2 inhibitors may enhance the anticancer efficacy for patients with high tumor ABCG2 level.

## Introduction

Multidrug resistance (MDR) is one of the major challenges in cancer therapy. The definition of MDR in cancer is the insensitivity of cancer cells to the neoplastic drugs (which has distinct chemical structures and mechanisms of action) despite earlier sensitivity to them. During exposure to anticancer drugs, cancer cells may develop MDR through decreasing drug uptake by downregulation of some membrane carriers or receptors, increasing drug efflux by upregulation of membrane transporters, and increasing the mutation of genes (Toyoda et al., 2019). Among all the mechanisms, overexpression of ABC transporters is considered as a key mediator of MDR (Wu et al., 2020c). Substantial studies suggest that ABCB1, ABCG2, and ABCC1 are the major MDR-related ABC transporters (Xiao et al., 2021). Specifically, ABCG2 transporter can mediate the efflux of a broad range of anticancer drugs and render cancer cells resistance to these drugs (Yang et al., 2020). ABCG2 was firstly discovered by Doyle et al. (1998) in a doxorubicin-resistant MCF-7/AdVp300 cells and was named Breast Cancer Resistance Protein (BCRP). The cDNA of ABCG2 was later isolated and characterized by Miyake et al. (1999) from a mitoxantrone-resistant S1-M1-80 cells and it was also name Mitoxantrone Resistance Gene (MXR). ABCG2 exerts its substrate efflux function using the energy from ATP hydrolysis catalyzed by the membrane-bound ATPase of ABCG2. Recent studies suggest that ABCG2 transports its substrate via a closed-to-open switch (Orlando and Liao, 2020). Upon binding of substrate drugs, ABCG2 will shift toward the inward-facing conformation. Subsequently, ATP binding facilitates the transport of substrate drugs from the cells to the extracellular space. To dates, a lot of chemotherapeutic agents and Tyrosine Kinase Inhibitors (TKI) are identified as substrates of ABCG2, including irinotecan (Fan et al., 2019), doxorubicin (Sáfár et al., 2021), imatinib (Kosztyu et al., 2014), tivantinib (Wu et al., 2020b), milciclib (Martínez-Chávez et al., 2021). Numerous clinical data have indicated ABCG2 expression level is correlated with the response to ABCG2 substrate drugs. For example, a good response to methotrexate is associated with a decrease in ABCG2 expression in patients with rheumatoid arthritis (Muto et al., 2021). Wang et al. (2021) reported that ABCG2 rs1871744 and rs4148157 genotype were significantly associated with the poor response in cancer patients receiving platinum-based chemotherapy. Palshof et al. (2020) found that colorectal cancer patients with low ABCG2 expression level had a higher chance of obtaining objective response when receiving irinotecan-based treatment. Hence, identifying anticancer drugs that are ABCG2 substrates can be beneficial to the development of clinical treatment strategy and to monitor the occurrence of drug resistance.

MLN7243 is a Ubiquitin-Activating Enzyme (UAE) inhibitor reported by Hyer et al. (2018). Ubiquitination is a post-translational medication pathway involved in wide range of cellular functions, including autophagy, immune response, and DNA damage response (Shaid et al., 2013). During ubiquitination, ubiquitin protein is attached to target proteins through catalysis pathway mediated by E1 ubiquitin-activating enzymes, E2 ubiquitin-conjugating enzymes and E3 ubiquitin ligases (Liu et al., 2021). UAE and UBA6, both referred as E1 enzymes, are known to initiate ubiquitin conjugation by regulating cellular ubiquitin-charging (Jin et al., 2007). As a first-in-class UAE inhibitor, MLN7243 potently inhibits the activity of UAE by forming MLN7243-ubiquitin adduct. Therefore, the drug can cause depletion of cellular ubiquitin conjugates, thereby disrupting cellular signaling pathways, inducing proteotoxic stress, and increasing DNA damage stress. MLN7243 produced substantial anticancer effect in a large number of solid tumor xenograft models (Hyer et al., 2018). In addition, a preclinical study suggested that MLN7243 has anticancer efficacy against Acute Myeloid Leukemia (AML) (Barghout et al., 2019). The study also identified missense mutation of UAE can confer AML cells resistance to MLN7243. Recent studies also suggest that MLN7243 is more effective in Schlafen11 deficient tumors (Murai et al., 2021). In leukemia cell lines, MLN7243 produced a stronger inhibition of ubiquitylation in Schlafen11-knockout cells than the wild-type cells. To dates, MLN7243 is in phase 1 clinical trial to investigate its anticancer effect in patients with AML or CML (NCT03816319). Previously, we identified another E1 enzyme inhibitor MLN4924 as a ABCG2 substrate (Wei et al., 2020). Therefore, it is intriguing to investigate whether other E1 enzyme inhibitors sharing similar chemical structures or mechanisms of action are substrates of ABCG2 transporter.

In the current study, we demonstrated that the intracellular accumulation and cytotoxicity of MLN7243 were significantly reduced in ABCG2-overexpressing cells. The inclusion of ABCG2 inhibitor was able to sensitize the drug-resistant cells to MLN7243, thereby reversing the drug resistance. Taken together, the combinational treatment of MLN7243 with ABCG2 inhibitor may produce better therapeutic response in tumors with high ABCG2 level.

## Materials and Methods

### Reagents

MLN7243 was provided by ChemieTek (Indianapolis, IN). Fumitremorgin C (FTC) was a gift from Dr. Susan Bates (Columbia University, NY). All the drug stock solutions were dissolved in DMSO. The reagents were purchased from Sigma Chemical Co (St. Louis, MO) unless stated otherwise.

### Cell Lines and Cell Culture

Cell culture was performed as stated previously (Zhang et al., 2020). Briefly, all cells were cultured in DMEM with 10% FBS and antibiotic. ABCG2-overexpressing cancer cells NCI-H460/TPT10 and S1-M1-80 were maintained in 10 μM of topotecan and 80 μM of mitoxantrone, respectively (Miyake et al., 1999; Lei et al., 2020). ABCG2 knockout cells and HEK293 gene-transfected cells were maintained in 5 and 2 mg/mL geneticin, respectively.

### Cytotoxicity Assay

The cytotoxicity of anticancer drugs was determined using MTT assay (Wu et al., 2020d). Cells were seeded into 96-well plates (5,000 cells/well) and allowed to attach overnight. Subsequently, serial concentrations of drugs were added to the designated wells. The 96-well plates were incubated for 72 h before data collection. At the last day, the MTT dye solution was applied and incubated for 4 h. The produced formazan crystals were dissolved in DMSO and the OD_750_ value was read.

### ABCG2 ATPase Assay

The ATP hydrolysis activity of ABCG2 ATPase was evaluated using PREDEASY ATPase Kits (TEBU-BIO nv, Boechout, Belgium) with modified protocols (Wu et al., 2019). Membrane vesicles were incubated in assay buffer, followed by the addition of MLN7243. The reaction was initiate by adding 5 mM ATP solution and terminated by adding SDS solution. The ATPase activity was measured by colorimetric method and calculated using the difference of the produced Pi with and without Na_3_VO_4_, which inhibits plasma membrane ATPases including Na^+^/K^+^-ATPase (Chifflet et al., 1988).

### Western Blot Analysis

The protein level of ABCG2 was determined by Western blot. The primary antibodies used were mouse monoclonal anti-ABCG2, and anti-GAPDH (1:1,000 dilution, Thermo Fisher Scientific Inc., Waltham, MA). HRP-conjugated secondary antibody was purchased from CST (Cell Signaling Technology Inc., Danvers, MA). The protein bands were visualized by ECL kit (Thermo Fisher Scientific Inc., Waltham, MA). The signals were analyzed by ImageJ software (NIH, MD) and ABCG2 expression levels were normalized to GAPDH before comparison.

### MLN7243 Accumulation Assay

The HPLC assay was carried out as previous described (Wu et al., 2021a). Cells were seeded into 6-well plates (200,000 cell/well) and incubated for 48 h. Cells were incubated in plain DMEM median with 20 μM of MLN7243 with or without 5 μM of ABCG2 inhibitor Ko143 for 2 h. Thereafter, cells were lysed and harvested with 0.5% SDS and acetonitrile. The supernatant was collected by centrifuging at 14,000 rpm for 10 min. HPLC purification and analysis were performed using the Agilent Technologies instrument (1,200 series) and monitored using a 1,100 series detector (254 nm). The eluents used were, water (solvent A) and methanol (solvent B), both solvents supplemented with 0.1% formic acid to maintain buffer capacity. Purification was done using a C18 analytical column (Agilent Eclipse Plus 2 mm, 4.5 × 250 mm). Flow rate: 1 mL/min. Gradient solvent system: 60:40 water: methanol to 2:98 water: methanol and the run time was 15 min. The standard curve was plotted using different concentrations of MLN4924 against the area under the curve.

### [^3^H]-Substrate Accumulation Assay

[^3^H]-mitoxantrone (Moravek Biochemicals Inc., Brea, CA) was used as a substrate drug to detect the intracellular drug accumulation in NCI-H460 and NCI-H460/TPT10 cells. Briefly, cells were seeded into 24-well plates (100,000 cells/well) and allowed to attach overnight. At the following day, cells were pretreated with 3 or 10 μM of MLN7243 or positive inhibitor Ko143. Subsequently, cells were incubated in medium with 5 nM of [^3^H]-mitoxantrone with or without MLN7243 or Ko143 for 2 h. After incubation, samples were collected, and the radioactivity were determined by Packard TRICARB 1900CA liquid scintillation analyzer (Packard Instrument, Downers Grove, IL).

### Docking Analysis

The detailed protocol of *in silico* docking analysis was carried out as stated previously (Trott and Olson, 2010; Wang et al., 2019). The protein model (PDB: 6VXI) selected is inward-facing with a resolution of 3.7 Å (Orlando and Liao, 2020). Preparation of ligand/receptor and the simulation were carried out with default settings. The top-scoring pose (sorted by affinity score: kcal/mol) was chose for final analysis and visualization.

### Data Analysis

All assays were run at least three times and all data were presented as mean ± SD. Data analysis was performed using One-way ANOVA in GraphPad software (Prism 8.1). Differences were considered statistically significant when ^∗^*P* < 0.05.

## Results

### The Cytotoxicity of MLN7243 in Parental and ABCG2-Overexpressing Cells

**FIGURE 1:**
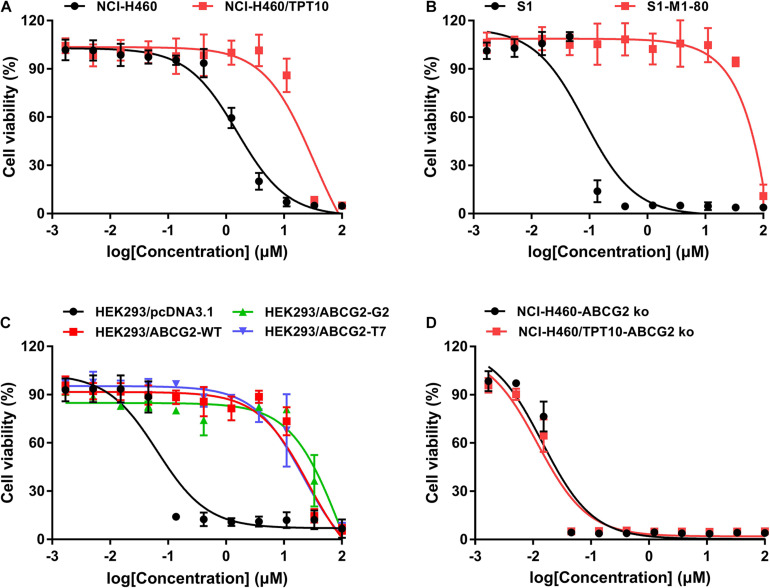
The cytotoxicity of MLN7243 in parental and drug-resistant cell lines. Cell viability curves for **(A)** NCI-H460 and NCI-H460/TPT10 cells, **(B)** S1 and S1-M1-80 cells, **(C)** HEK293/pcDNA3.1 and HEK293/ABCG2-WT, -R482G, -R482T cells, and **(D)** NCI-H460-ABCG2 ko and NCI-H460/TPT10-ABCG2 ko cells. Data are expressed as mean ± SD from a representative of three independent experiments.

The cytotoxicity of MLN7243 was determined in multiple pairs of parental and ABCG2-overexpressing cell lines. Here, we used the human non-small cell lung cancer NCI-H460 and its topotecan-selected NCI-H460/TPT10 subline, human colon cancer S1 and its mitoxantrone selected S1-M1-80 subline, as well as HEK293 cells stably transfected with an empty pcDNA3.1 vector or pcDNA3.1 vectors containing full-length wild-type (WT) or mutant-*ABCG2*. In addition, the *ABCG2* knockout cell lines NCI-H460-ABCG2 ko and NCI-H460/TPT10-ABCG2 ko were used for validation. The cell viability curves, and the calculated IC_50_ values were summarized and presented in Figure 1 and Table 1. Our results showed that all ABCG2-overexpressing cells were significantly less sensitive to MLN7243 than the parental cells, as indicated by the gap between the cell viability curves. In NCI-H460/TPT10 and S1-M1-80 cells, the Resistance-Fold (RF) were 23-fold and over 1,000-fold, respectively. Similarly, HEK293 cells overexpressing WT- or mutant-ABCG2 were highly resistant to MLN7243, with more than 500-fold resistance. Subsequently, when the ABCG2-overexpressing cells were co-incubated with selective ABCG2 inhibitor Ko143, the RF were significantly decreased. For instance, the RF value decreased from over 1,000- to 0.83-fold in S1-M1-80 cells and decreased from more than 500-fold to around 2-fold in the HEK293/ABCG2 cells, suggesting a complete reversal of drug resistance. In contrast, Ko143 did not significantly affect the cytotoxicity of MLN7243 in S1 and HEK293 cells, suggesting that MLN7243 resistance is primarily attributed to ABCG2 overexpression in these MDR cell lines. In addition, the combination of MLN7243 with another ABCG2 inhibitor FTC showed similar trends (data not shown). The results suggest that ABCG2 inhibition is able to enhance the sensitivity of drug-resistant cells to MLN7243. Surprisingly, the IC_50_ was significantly decreased when NCI-H460 cells were co-incubated with Ko143. It is possible that the endogenous ABCG2 expression in NCI-H460 cells can confer resistance to MLN7243. Therefore, MTT assay using the *ABCG2* knockout NCI-H460-ko and NCI-H460/TPT10-ko cells were performed to further verify our finding. As shown in Figure 1D, upon *ABCG2* knockout, the drug-resistant cells restored the sensitivity to MLN7243, and the cell viability curve was overlapping with that of the parental cells. Furthermore, the IC_50_ of MLN7243 in both knockout cells were comparable to that in the parental cells co-incubated with Ko143. These results suggest that MLN7243 may be a potent ABCG2 substrate, which leads to its decreased cytotoxicity in ABCG2-overexpressing cells.

**TABLE 1:** The cytotoxicity of MLN7243 in cells overexpressing the ABCG2 transporter.

Treatment	IC_50_ value ± SD^a^ (μM, resistance fold^b^)	
	
	MLN7243	MLN7243 + Ko143 3 μM
NCI-H460	1.476 ± 0.430(1.00)	0.014 ± 0.002(0.009)*
NCI-H460/TPT10	34.070 ± 6.826(23.08)*	0.012 ± 0.006(0.008)*
S1	0.084 ± 0.007(1.00)	0.103 ± 0.021(1.23)
S1-M1-80	>100 (> 1,000)^∗^	0.070 ± 0.004(0.83)
HEK293/pcDNA3.1	0.045 ± 0.007(1.00)	0.037 ± 0.010(0.82)
HEK293/ABCG2-WT	33.197 ± 6.779(737.3)*	0.029 ± 0.014(0.64)
HEK293/ABCG2-R482G	50.833 ± 18.451(> 1,000)*	0.274 ± 0.149(6.09)*
HEK293/ABCG2-R482T	25.555 ± 10.025(567.9)*	0.108 ± 0.008(2.40)
NCI-H460-ABCG2 ko	0.015 ± 0.003(1.00)	0.013 ± 0.005(0.87)
NCI-H460/TPT10-ABCG2 ko	0.012 ± 0.001(0.86)	0.012 ± 0.005(0.80)

*^*a*^IC_50_ values are represented as mean ± SD of at least three independent experiments.*

^*b*^Rf, Resistance fold was calculated by dividing the IC_50_ values of substrates in the presence or absence of inhibitor by the IC_50_ of parental cells without inhibitor.

*P < 0.05 vs. the control group without Ko143.

### MLN7243 Stimulated ABCG2 ATPase Activity

**FIGURE 2:**
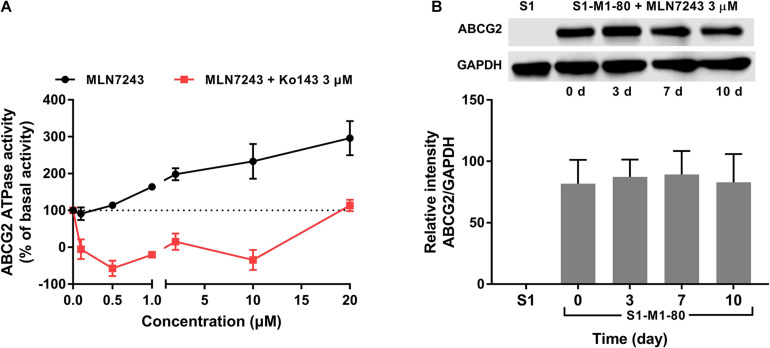
MLN7243 stimulated ABCG2 ATPase activities without affecting to the protein expression level. **(A)** The effect of MLN7243 on ABCG2-mediated ATPase activity at concentration range from 0 to 20 μM. Ko143 was used as an ABCG2 ATPase inhibitor. **(B)** The effect of MLN7243 on the expression level of ABCG2 in S1-M1-80 cells after 10 d treatment. Data are expressed as mean ± SD derived from three independent experiments. *p < 0.05 vs. the control group.

To further verify if MLN7243 is an ABCG2 substrate, the ATPase assay was carried out to explore the interaction between MLN7243 and ABCG2. As shown in Figure 2A, MLN7243 concentration-dependently induced the ATPase activity of ABCG2. The maximum stimulation folds were 2.96-fold over basal level with EC_50_ at 1.78 μM (as indicated by the black curve). While in the red curve, Ko143 significantly inhibited the activity of ABCG2 ATPase as compared with the basal level. With the increasing concentrations of MLN7243, the Ko143-related inhibition was gradually diminished, and the ATPase activity was restored to the basal level when adding 20 μM of MLN7243. Therefore, the data suggests that MLN7243 can bind to the substrate-binding site, thereby facilitating the binding and hydrolysis of ATP.

### MLN7243 Did Not Upregulate ABCG2 Protein Expression Level in ABCG2-Overexpressing Cancer Cells

As we characterized MLN7243 as a substrate of ABCG2, cancer cells may express higher level of ABCG2 protein upon MLN7243 treatment. To this end, Western blotting was performed to further evaluate whether MLN7243 can upregulate the expression level of ABCG2 in the drug-resistant cells. As shown in Figure 2B, the drug-resistant S1-M1-80 cells were incubated with 3 μM of MLN7243 for up to 10 consecutive days. However, no significant difference was observed within the treatment period. This is not surprising as cancer cells with the low endogenous expression of ABCG2 is sufficient to confer resistance to MLN7243.

### The Accumulation of MLN7243 Was Decreased in ABCG2-Overexpressing Cells

**FIGURE 3:**
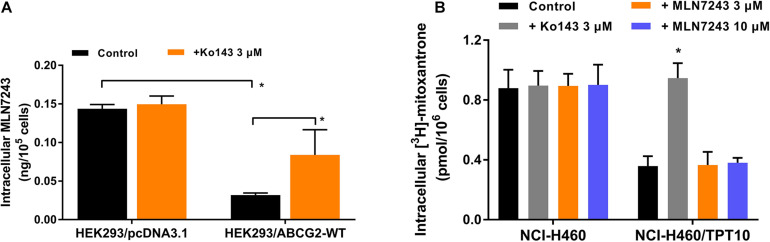
MLN7243 was transported out from ABCG2-overexpressing cells but did not competitively inhibit the efflux of another ABCG2 substrate. **(A)** The intracellular accumulation of MLN7243 in HEK293/pcDNA3.1 and HEK293/ABCG2-WT determined by HPLC assay. **(B)** The intracellular accumulation of [^3^H]-mitoxantrone in NCI-H460 and NCI-H460/TPT10 cells after co-incubated with 3 or 10 μM of MLN7243. Data are expressed as mean ± SD derived from three independent experiments *p < 0.05 vs. the control group.

Direct measurement of intracellular MLN7243 concentration can provide another strong evidence to confirm whether MLN7243 is a substrate of ABCG2. Consequently, HPLC assay was performed to quantify the accumulation of MLN7243 in HEK293/pcDNA3.1 and HEK293/ABCG2-WT cells. With overexpression of ABCG2 as the major difference between the two HEK293 cells, the results may be more accurate compared to the drug selected cancer cells which may have multiple MDR-related factors. As shown in Figure 3A, the accumulation of MLN7243 was significantly lower in HEK293/ABCG2-WT cells compared to the HEK293/pcDNA3.1 cells, suggesting ABCG2 can actively extrude MLN7243 to the extracellular space from the cells. Furthermore, when HEK293/ABCG2-WT cells were co-incubated with Ko143, the intracellular concentration of MLN7243 was significantly increased. Therefore, the results further confirmed that MLN7243 is an ABCG2 substrate.

### MLN7243 Did Not Increase the Accumulation of Mitoxantrone, an ABCG2 Substrate Drug

To further evaluate the substrate property of MLN7243, [^3^H]-mitoxantrone accumulation assay was performed. As a substrate of ABCG2, MLN7243 may compete with other substrates for the drug-binding site of ABCG2 transporter, resulting in decreased transportation of substrates. By measuring the intracellular accumulation of mitoxantrone, it allows the evaluation of whether MLN7243 can competitively inhibit the efflux of mitoxantrone mediated by ABCG2 and thereby serving as a potential MDR reversal agent. As shown in Figure 3B, the accumulation of mitoxantrone was significantly attenuated in NCI-H460/TPT10 cells, suggesting a large portion of mitoxantrone was pumped out from the cells by ABCG2 transporter. However, neither Ko143 or MLN7243 treatment groups affected to the [^3^H]-mitoxantrone accumulation level in NCI-H460 cells. In contrast, Ko143, but not MLN7243, significantly elevated the intracellular accumulation of [^3^H]-mitoxantrone in NCI-H460/TPT10. These results suggest that MLN7243 may not compete with other ABCG2 substrates for the drug-binding site.

### Docking Study of MLN7243 With ABCG2 Protein Model

**FIGURE 4:**
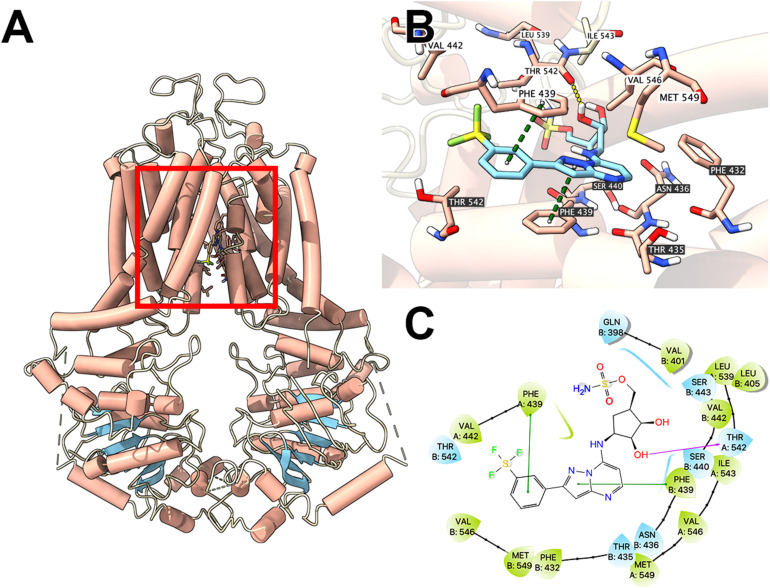
Interaction between MLN7243 and human ABCG2 protein model. **(A)** Overview of the best-scoring pose of MLN7243 in the drug binding pocket of ABCG2 protein (6VXI). ABCG2 was displayed as colored tubes (helix: red; strand: blue; coil: white). MLN7243 was displayed as colored sticks. Carbon: cyan; oxygen: red; nitrogen: blue, hydrogen: white. **(B)** Details of the interaction between MLN7243 and ABCG2 binding pocket. ABCG2 helices were displayed as colored tubes (helix: red; strand: blue; coil: white). Important residues were displayed as colored sticks (carbon: red; oxygen: red; nitrogen: blue; hydrogen: white). MLN7243 was displayed as colored sticks (same as in A). Residues in chain A and B was labeled on white and black background, respectively. Hydrogen bonds were displayed as yellow dash lines. π−π stacking interactions were displayed as green dash lines. **(C)** 2D diagram of the interaction between MLN7243 and ABCG2 binding pocket. Important amino acids within 3 Å from the ligand were displayed as colored bubbles (green: hydrophobic; blue: polar). Purple solid lines with arrow indicate hydrogen bonds. Green solid lines indicate π−π stacking interactions.

To illustrate potential binding pattern between MLN7243 and the ABCG2 transporter, computational docking analysis was performed. MLN7243 docked into the ABCG2 binding site with a high affinity score of -9.553 kcal/mol. Details of ligand-receptor interaction was depicted in Figure 4. When docked into the substrate-binding site, MLN7243 was stabilized by two π-π stacking interactions with Phe439 in both chains A and B. A hydrogen bond was also identified between MLN7243 and Thr542 in chain A.

## Discussion

MDR is one of the inevitable challenges in cancer treatment, especially in conventional chemotherapy and targeted therapy. When cancer cells develop MDR phenotype, it becomes insensitive to a broad range of anticancer drugs, which may lead to treatment failure (Juan-Carlos et al., 2021). Importantly, overexpression of ABCG2 transporter is characterized as a major mechanism that regulates MDR in cancer cells. Belonging to the ABC transporter superfamily, ABCG2 is known for its efflux function which actively pump a wide variety of substrates into the extracellular space (Lusvarghi et al., 2020). *In vitro* and *in vivo* data has suggested that polymorphisms and expression level of ABCG2 may affect to the pharmacokinetics of substrate drugs (Mao and Unadkat, 2015; Zhang et al., 2018). Therefore, it is important to explore the potential interaction between anticancer drugs and ABCG2 transporter.

In the current study, we demonstrated that MLN7243, a UAE inhibitor currently in clinical trial, is a potent substrate of ABCG2. As suggested by The International Transporter Consortium (ITC), several methods can be utilized to explore the interaction between drugs and transporters (Giacomini et al., 2010). These include membrane-based assays such as ATPase assay, cell-based assays that measure the uptake or efflux of substrate drugs, and *in vivo* models to evaluate the pharmacokinetics of the compounds. Here, we first performed MTT assay to compare the cytotoxicity of MLN7243 in parental and ABCG2-overexpressing cells. Both ABCG2-overexpressing cancer cells NCI-H460/TPT10 and S1-M1-80 were significantly insensitive to MLN7243, and S1-M1-80 cells showed more than 1,000-fold resistance to MLN7243. Since the drug-selected MDR cancer cells may develop multiple mechanisms to exhibit MDR phenotype, HEK293 cells transfected with *ABCG2* gene were used to validate our finding. Similarly, both WT- and mutant-ABCG2-transfectant cell lines showed significant resistance to MLN7243. In addition, previous studies have suggested that the mutation at residue 482 may affect to the substrate binding and efflux behavior. Particularly, WT-ABCG2 does not transport lipophilic antifolate while the mutant Gly482 and Thr482 variants are unable to transport methotrexate (Chen et al., 2003; Bram et al., 2006). Our MTT data showed that the cytotoxicity of MLN7243 was decreased in both WT- and mutant-ABCG2 overexpressing cells, suggesting the R482 mutation may not affect to the transportation of MLN7243. Furthermore, when the ABCG2-overexpressing cells were co-incubated with Ko143 and MLN7243, a complete reversal of drug resistance was observed. To determine whether other ABCG2 inhibitor can produce the similar effect, we used another well-established ABCG2 inhibitor, FTC, to carry out the combinational treatment (Data not shown). The results showed that FTC was also able to complete reverse the drug resistance in ABCG2-overexpressing cells without affecting the cytotoxicity of MLN7243 in the parental cells. Hence, the inhibition of ABCG2 efflux function can significantly increase the sensitivity of MDR cells to MLN7243, suggesting MLN7243 is a transported substrate of ABCG2. Interestingly, the combination treatment was also effective in parental NCI-H460 cells but not in S1 or HEK293/pcDNA3.1 cells. It is most likely that the endogenous expression of ABCG2 in NCI-H460 cells produces intrinsic MLN7243 resistance to some extent. The MTT results obtained from *ABCG2* knockout cells showed that both NCI-H460 and NCI-H460/TPT10 cells became more sensitive to MLN7243 after *ABCG2* knockout. In addition, the effect of *ABCG2* knockout was similar to the functional inhibition using Ko143. Therefore, our results provided strong evidence to show MLN7243 is a substrate of ABCG2.

As an ATP-dependent transporter, ATP hydrolysis is necessary for the efflux function of ABCG2. The ATPase assay showed that MLN7243 can concentration-dependently induce the activity of ABCG2 ATPase. In addition, with the increasing concentration of MLN7243, it antagonized the inhibitory effect of Ko143 and restored the ABCG2 ATPase activity to basal level. Hence, the ATPase assay confirmed that MLN7243 may act as a substrate of ABCG2 and thereby stimulating the efflux function of ABCG2. Subsequently, we performed the HPLC assay to directly assess the intracellular accumulation of MLN7243 in parental and drug-resistant cells. It is postulated that, as a substrate drug, MLN7243 will accumulate less in drug-resistant cells than in the parental cells. Our data showed that the concentration of MLN7243 was around fourfold lower in HEK293/ABCG2-WT cells as compared to the HEK293/pcDNA3.1 cells. Since the major difference between the two HEK293 cells was the overexpressing of ABCG2, the decreased accumulation of MLN7243 in HEK293/ABCG2-WT cells can be attributed to active transport by the ABCG2 transporter. In summary, our *in vitro* data provided direct evidence to show that MLN7243 is a substrate of ABCG2, and the anticancer efficacy is significantly attenuated in ABCG2-overexpressing cells.

To further evaluate the interaction between MLN7243 and ABCG2 transporter, we performed Western blotting and [^3^H]-mitoxantrone accumulation assay. Previous studies have reported that some substrate drugs can induce the protein expression of ABCG2 after short-term or long-term exposure (Wu et al., 2020b, 2021b). To this end, we treated the drug-resistant cells with MLN7243 for up to 10 days. However, no significant alteration in ABCG2 expression level was observed. This may be because the cells were already highly resistant to MLN7243, making the induction effect negligible. Whether MLN7243 can induce the overexpression of ABCG2 in parental cells was not determined in this experiment and should be further investigate in future study. Another potential property of substrate drug is the competitive inhibitory effect. It is suggested that some ABCG2 substrates such as gefitinib (Chen et al., 2011), GSK1070916 (Wu et al., 2021a) can inhibit the efflux of another substrate drug and thereby reverse the drug resistance. [^3^H]-mitoxantrone accumulation assay was performed to explore this interaction. Our results showed that MLN7243 did not affect to the intracellular accumulation of mitoxantrone in parental or ABCG2-overexpressing cells. In contrast, the positive ABCG2 inhibitor Ko143 was able to increase the intracellular accumulation of mitoxantrone in ABCG2-overexpressing cells to the similar level as that in the parental cells. Since ABCG2 transporter may have multiple substrate-binding site (Clark et al., 2006; Cox et al., 2018), it is possible that MLN7243 and mitoxantrone may have distinct binding position or MLN7243 is insufficient to compete with another substrate for the drug-binding site. The in-depth mechanisms remain to be determined.

The computational docking analysis can be utilized to facilitate the understanding of ligand-transporter interactions. Although the predicted results may not represent the real-world situation, such computer-aided tool has been applied in screening substrates of ABC transporters (Jackson et al., 2018). Learning that MLN7243 is an ABCG2 substrate, the *in silico* docking analysis was performed using the recently reported cryo-EM structures of human ABCG2. The predicted docking score was −9.553 kcal/mol, which is similar to other substrates such as OTS964 (Yang et al., 2021) and samotolisib (Wu et al., 2020a). Hence, the result indicates that MLN7243 may interact with ABCG2 in the substrate-binding site.

In conclusion, our study revealed that MLN7243 is a potent substrate of ABCG2. Therefore, the therapeutic effect of MLN7243 may be attenuated in patients with high tumor ABCG2 expression. It should be noted that other MDR-related ABC transporters such as ABCB1 and ABCC1 also actively mediate the drug resistance in cancer treatment. The interaction between MLN7243 and these ABC transporters remain inconclusive and should be evaluated. Future studies may also focus on validating the results *in vivo* and developing effective strategies to overcome the ABCG2-mediated drug resistance.

## Data Availability Statement

The original contributions presented in the study are included in the article/supplementary material, further inquiries can be directed to the corresponding author/s.

## Author Contributions

Z-XW, LZ, and Z-SC: conceptualization, writing – review, and editing. Z-XW, YY, J-QW, SN, Z-NL, Q-XT, and LZ: methodology. Z-XW and LZ: writing – original draft preparation. LZ and Z-SC: supervision. All authors have read and agreed to the published version of the manuscript.

## Conflict of Interest

The authors declare that the research was conducted in the absence of any commercial or financial relationships that could be construed as a potential conflict of interest.
